# Bile Acid Signaling in Liver Metabolism and Diseases

**DOI:** 10.1155/2012/754067

**Published:** 2011-10-03

**Authors:** Tiangang Li, John Y. L. Chiang

**Affiliations:** Department of Integrative Medical Sciences, Northeast Ohio Medical University, Rootstown, Ohio, OH 44272, USA

## Abstract

Obesity, diabetes, and metabolic syndromes are increasingly recognized as health concerns worldwide. Overnutrition and insulin resistance are the major causes of diabetic hyperglycemia and hyperlipidemia in humans. Studies in the past decade provide evidence that bile acids are not just biological detergents facilitating gut nutrient absorption, but also important metabolic regulators of glucose and lipid homeostasis. Pharmacological alteration of bile acid metabolism or bile acid signaling pathways such as using bile acid receptor agonists or bile acid binding resins may be a promising therapeutic strategy for the treatment of obesity and diabetes. On the other hand, bile acid signaling is complex, and the molecular mechanisms mediating the bile acid effects are still not completely understood. This paper will summarize recent advances in our understanding of bile acid signaling in regulation of glucose and lipid metabolism, and the potentials of developing novel therapeutic strategies that target bile acid metabolism for the treatment of metabolic disorders.

## 1. Introduction

Bile acids are produced only in the liver as the end products of cholesterol catabolism [1, 2]. In addition to the classic function of bile acids in facilitating hepatobiliary secretion of endogenous metabolites and xenobiotics and intestine absorption of lipophilic nutrients, bile acids also play equally important roles in controlling the metabolism of glucose and lipids in the enterohepatic system, and energy expenditure in peripheral tissues [3, 4]. Because of such a close association between bile acid signaling and metabolic homeostasis, targeting bile acid metabolism by using bile acid receptor agonists or bile acid-binding resins have proven to be effective in improving lipid and glucose homeostasis in obesity and diabetes [5]. Furthermore, stimulating de novo bile acid synthesis prevented, whereas, disruption of bile acid signaling caused insulin resistance and dyslipidemia in mice, indicating that impaired bile acid homeostasis may likely contribute to the pathogenesis of metabolic disorders [6–9]. This paper will summarize recent advances in our understanding of bile acid signaling regulation of glucose and lipid metabolism and the potentials of developing novel therapeutic strategies that target bile acid metabolism for the treatment of metabolic disorders.

## 2. Bile Acid Synthesis

Bile acids are the end product of cholesterol catabolism in the liver [1, 10–12]. In humans, the bile acid pool consists of primary bile acids cholic acid (CA), chenodeoxycholic acid (CDCA), secondary bile acids deoxycholic acid (DCA), and lithocholic acid (LCA). Primary bile acids are synthesized from cholesterol through two general pathways, the classic pathway and the alternative pathway. Secondary bile acids are derived from primary bile acids in the intestine by bacterial enzymes. Enzymes that catalyze these multistep reactions are located in the endoplasmic reticulum, mitochondria, cytosol, and peroxisomes. In humans, the classic pathway is considered as the major bile acid synthetic pathway (accounts for more than 90% of total bile acid production) and produces CA and CDCA in approximately equal amounts. Cholesterol 7*α*-hydroxylase (CYP7A1), a microsomal cytochrome p450 enzyme, catalyzes the first and rate-limiting step in the classic pathway [13]. The intermediate product in the classic pathway 7*α*-hydroxy-4-cholestene-3-one serves as the common precursor for both CA and CDCA. 7*α*-hydroxy-4-cholestene-3-one can be hydroxylated at C-12 position by microsomal sterol 12*α*-hydroxylase (CYP8B1), followed up by several reactions including mitochondrial 27-hydroxylase (CYP27A1) to cleave a 3-carbon unit and eventually converted to CA. Without 12*α*-hydroxylation, 7*α*-hydroxy-4-cholestene-3-one is converted to CDCA. Thus, CYP7A1 controls the overall rate of bile acid production, while CYP8B1 controls the CA: CDCA ratio in the bile acid pool. The alternative pathway (also known as acidic pathway), which is thought to account for less than 10% of the total bile acid synthesis in humans, mainly produces CDCA. CYP27A1 catalyzes hydroxylation of cholesterol to 27-hydroxycholesterol and then 3*β*-dihydroxy-5-cholestionic acid [14]. Oxysterol 7*α*-hydroxylase (CYP7B1) then catalyzes the hydroxylation reaction at C-7 position of these two intermediates, which are subsequently converted to CDCA by the same enzymes in the classic pathway.

## 3. Bile Acid Transport and Enterohepatic Circulation

**Figure 1:**
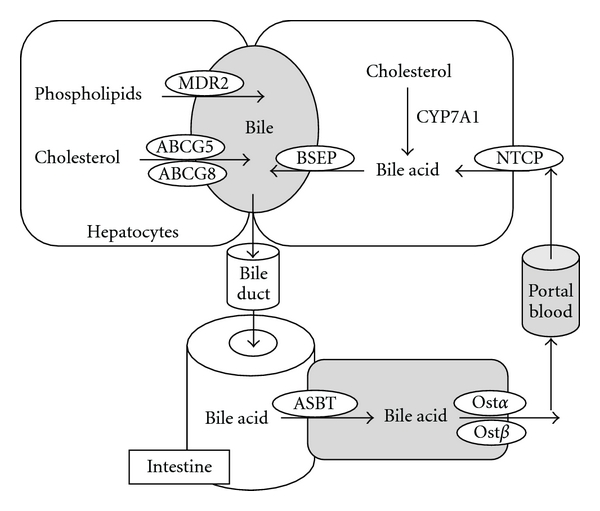
Enterohepatic circulation of the bile. Bile acids are synthesized from cholesterol in the hepatocytes. CYP7A1 regulates the rate-limiting step in the classic bile acid biosynthetic pathway. Bile acids are secreted into the gallbladder via BSEP. Phospholipids are transported via MDR2, and cholesterol is transported by the ABCG5/G8 transporters into the bile. In the gallbladder, bile acids, phospholipids, and cholesterol form mixed micelles to solubilize cholesterol and to reduce bile acid toxicity. After meal intake, gallbladder releases bile into the small intestine where bile acids facilitate the absorption of dietary lipids and vitamins. At the terminal ileum, most of the bile acids are reabsorbed by ASBT into the enterocytes, and secreted into the portal circulation via basolateral bile acid transporters Ost*α*/Ost*β*. At the basolateral membrane of the hepatocytes, bile acids are taken up by the NTCP transporter for resecretion into the gallbladder.

Bile acids, once produced in the liver, are transported across the canalicular membrane of the hepatocytes into the bile and stored in the gallbladder. After each meal, gallbladder bile acids are released into the intestinal tract, efficiently reabsorbed in the ileum, and transported back to the liver via portal blood for reexcretion into the bile. This process is referred to as enterohepatic circulation of bile acids [10] (Figure 1). Bile acid transporters play important roles in this transport process [15]. The biliary excretion of bile acids is the major driving force of bile flow. The bile acid pool size is defined as the total amount of bile acids circulating in the enterohepatic circulation. In humans, bile acid pool consists of CA, CDCA, and DCA in an approximate 40 : 40 : 20 ratio. In mice, the majority of the CDCA is converted into muricholic acids (MCAs), which are highly soluble and less toxic.

Hepatocytes are polarized epithelial cells with basolateral (sinusoidal) and apical (canalicular) membrane domains. Hepatocytes take up bile acids through the basolateral membrane, which is in direct contact with the portal blood plasma, and excrete bile acid at the canalicular membrane into the bile [16]. Since the biliary bile acids concentration is about 100- to 1000-fold higher in the bile than in the hepatocytes, canalicular bile acid transport represents the rate-limiting step in bile formation. Several members of the ATP-binding cassette (ABC) transporter family are responsible for transporting bile acids and other organic compounds across the canalicular membrane against their concentration gradients. The bile salt export pump (BSEP, ABCB11), originally identified as the sister of P-glycoprotein (SPGP), is mainly responsible for bile acid transport at the canalicular membrane [17] (Figure 1). Mutations in *BSEP* were first identified in patients with progressive familial intrahepatic cholestasis subtype 2 (PFIC-2). The absence of functional BSEP in the hepatic canalicular membrane and less than 1% of normal biliary bile acid concentration found in these patients suggested that BSEP is the major canalicular bile acid transport system [18]. Phospholipids are excreted via the phospholipid flippase MDR2 (ABCB4) (Figure 1), and the major phospholipid in the bile is phosphatidylcholine [19, 20]. Biliary-free cholesterol secretion mediated by ABCG5/G8 transporters is an important route for hepatic cholesterol elimination. Bile acids, phospholipids, and cholesterol are three major organic solutes of the bile and once secreted, they form mixed micelles to increase cholesterol solubility and reduce their toxicity to the bile duct. Bile acids are conjugated with taurine or glycine in the peroxisomes and present as bile salts. They cannot cross the hepatocyte membrane and need active transport mechanisms for cellular uptake [21]. Two bile acid transporters, Na^+^-dependent taurocholate transporter (NTCP) (Figure 1) and organic anion transporters (OATPs) are responsible for basolateral bile acid transport into the hepatocytes. 

In the intestine, bile salts are deconjugated, and bacterial 7*α*-dehydroxylase removed a hydroxy group from C-7 and converts CA to DCA and CDCA to LCA. These secondary bile acids are highly toxic. In the intestine lumen, bile acids are reabsorbed mostly at the terminal ileum. Like the hepatic basolateral uptake system, intestinal bile acid uptake is also mainly mediated by the apical sodium-dependent bile salt transporter (ASBT) (Figure 1) [22]. Once absorbed into the enterocytes, bile acids bind the intestinal bile acid binding protein (I-BABP) and are transported to the basolateral membrane for secretion [23]. Recently identified heterodimeric organic solute transporters OST*α* and OST*β* appeared to be the major basolateral bile acid transport system in the intestine and many other epithelial cells (Figure 1) [24]. This is supported by studies showing that overexpression of OST*α* and OST*β* in mice enhanced basolateral efflux of taurocholate, while mice lacking *ost*α** showed marked decreases in intestinal bile acid absorption, serum bile acid concentration, and bile acid pool size.

## 4. Regulation of Bile Acid Synthesis

### 4.1. Bile Acid Feedback Regulation of Its Own Synthesis

It is clear that bile acid synthesis is mainly controlled via the transcriptional regulation of the *CYP7A1* gene [11]. It is well established that the *CYP7A1* gene is repressed by bile acids. This bile acid feedback repression mechanism allows the liver to efficiently increase or decrease bile acid synthesis in response to changes in bile acid levels and thus to maintain a constant bile acid pool. It is thought that bile acid feedback inhibition of *CYP7A1* is mediated by the bile acid-activated nuclear receptor farnesoid X receptor (FXR) in the hepatocytes and the enterocytes (Table 1). It was first discovered that hepatic FXR induced a negative nuclear receptor small heterodimer partner (SHP), which interacts with and represses the transcriptional activator liver-related homologue-1 (LRH-1) that binds to the *CYP7A1* gene promoter and thus inhibit *CYP7A1* transcription [25]. However, the repression of *CYP7A1* by bile acids and FXR agonist in SHP-deficient mice implies that FXR-SHP-LRH-1 cascade is not the only pathway mediating bile acid feedback inhibition of CYP7A1 and redundant pathways also exist [26, 27]. More recently, FXR was shown to induce intestine fibroblast growth factor 15 (FGF15) which may act as an endocrine hormone to repress *CYP7A1* gene transcription via ERK signaling activation [28]. Direct infusion of recombinant FGF15 into mouse blood circulation or overexpression of FGF15 in mouse livers via an adenovirus expression vector caused marked repression of CYP7A1 mRNA expression. The identification of an intestine-initiated endocrine mechanism in mediating bile acid feedback regulation is consistent with the fact of intestine being the major organ for bile acid reabsorption and retention. Such finding also provides an explanation to a long observed phenomenon that intraduodenal, but not intravenous, infusion of taurocholic acid repressed CYP7A1 mRNA expression in rats [29]. In mice lacking functional Ost*α*/Ost*β*, bile acid transport to the liver was reduced and bile acids accumulated in the intestine. Interestingly, these mice showed increased intestine FGF15 expression and reduced liver CYP7A1 mRNA and total bile acid pool [30]. Furthermore, intestine-specific FXR knockout, but not liver-specific FXR knockout, prevented GW4064 repression of liver CYP7A1 gene expression in mice [31]. These studies collectively suggest that intestine FXR plays a dominant role in mediating bile acid feedback repression of bile acid synthesis. Unfortunately, data on detection of the presence of FGF15 protein in the mouse circulation is lacking, and such evidence is needed in order to eventually establish the endocrine mechanism of feedback regulation of bile acid synthesis. Human FGF19 shares ~51% amino acid sequence identity with mouse FGF15 and is considered as the mouse FGF15 orthologue. FGF19 has been shown to repress *CYP7A1* in human hepatocytes [32]. In contrast to FGF15 that is not detectable in mouse livers and circulation, FGF19 mRNA is detectable in human livers and human hepatocytes [32, 33]. FGF19 protein is present in human circulation [34]. In human hepatocytes, FGF19 is highly inducible by bile acids or FXR agonists [32]. Since adenovirus-mediated overexpression of FGF15 in mouse liver has been shown to repress *CYP7A1*, it is likely that bile acid accumulation in human liver may induce FXR/FGF19 pathway to repress CYP7A1 in an autocrine manner [32]. Previous studies from us and others showed that bile acids were able to activate FXR-independent cell signaling pathways to repress the *CYP7A1* gene [35, 36]. We recently showed that feeding FXR knockout mice a cholic acid-containing diet still repressed CYP7A1 despite the absence of FGF15 or SHP induction [6]. These results indicate that in response to super-physiological concentrations of bile acids, redundant pathways are stimulated to repress bile acid synthesis. These pathways ensure prompt repression of de novo bile acid synthesis in response to elevated bile acid levels in the liver and/or the intestine.

### 4.2. Nutrient Effects on Hepatic Bile Acid Synthesis

Despite the majority of studies being focused on the regulatory roles of bile acids in nutrient metabolism, there is also evidence that nutrition could directly regulate bile acid synthesis. In humans, CYP7A1 activity, as determined by a serum surrogate 7alpha-hydroxycholest-4-en-3-one (C4), increases during postprandial periods during the day and decreases during fasting and at night [37]. A metabolomic study also identified bile acids as the most markedly elevated metabolites in human sera after an oral glucose challenge in patients with normal glucose tolerance, but this response was blunted in patients with impaired glucose tolerance [38]. Since postprandial period is the highly active in metabolism and humans undergo fasting-to-feeding cycles a few times a day, these observations could indicate an important link between bile acid synthesis and postprandial nutrient absorption and metabolism. Nutrient-activated signaling such as those by glucose or insulin is by far the most important signaling that regulates postprandial metabolism. Using primary human hepatocytes, we have demonstrated that insulin rapidly induced while glucagon repressed CYP7A1 mRNA [39, 40]. We also reported that glucose induced *CYP7A1* gene transcription via inducing histone hyperacetylation in *CYP7A1* gene chromatin [41]. Although our in vitro study provide mechanistic support for human observations, studies using in vivo mouse models yielded controversial results. It has been shown that PGC-1*α* acts as a coactivator of HNF4*α* and induces CYP7A1 during fasting in mice [42]. Furthermore, CYP7A1 mRNA was induced in STZ-treated type-I diabetic rats lacking insulin secretion [43], which has led to the speculation that insulin may repress *CYP7A1* gene in rats. On the other hand, more recent studies seem to be contradictory to these early observations. First, mouse CYP7A1 mRNA expression peaked during the early dark cycle when food intake was the most active [44, 45]. Furthermore, restricted feeding during light cycles shifted the peak of CYP7A1 mRNA expression from dark cycles to light cycles [44]. Such evidence seems to imply that liver bile acid synthesis and liver metabolism are coordinately controlled. Further studies are necessary to determine if nutritional regulation of bile acid synthesis may play a role in metabolic homeostasis during fasting to refeeding cycles.

## 5. Bile Acid Regulation of Glucose Metabolism

### 5.1. FXR and Glucose Metabolism

Diabetes is associated with impaired peripheral glucose clearance and increased hepatic glucose production during fasting, which lead to postprandial and fasting hyperglycemia. Initial evidence that bile acids may regulate glucose metabolism came from studies showing that FXR agonist induced phosphoenolpyruvate Carboxykinase (PEPCK) mRNA expression (Table 1) and glucose output in human and rat hepatocytes [46]. Treating mice with an FXR agonist also induced hepatic PEPCK mRNA expression in mice in vivo [46]. A FXR binding site has been identified in the promoter of PEPCK gene. In contrast, later studies carried out in *fxr* knockout mice revealed that FXR-deficient mice had insulin resistance and hyperglycemia phenotypes. Administration of a FXR agonist GW4064 decreased serum glucose, increased liver glycogen, and improved insulin sensitivity in diabetic *db/db* mice [7, 8]. A number of recent studies showed that bile acids and FXR repressed hepatic *PEPCK* and *G6Pase* gene expression and thus liver gluconeogenesis. In this case, it is shown that bile acids may induce the repressor SHP, which inhibits *PEPCK* via inhibiting C/EBP [47], FoxO1 [48], and Glucocorticoid receptor [49]. Although these liver effects of FXR activation may prevent fasting hyperglycemia, it does not sufficiently explain the increased insulin sensitivity and glucose disposal in FXR agonist-treated mice as determined by glucose and insulin tolerance tests. In a similar study, Cariou et al. used hyperinsulinemic euglycemic clamp and demonstrated that FXR-deficiency is associated with decreased whole-body glucose disposal, suggesting a role of FXR in regulating peripheral glucose metabolism [50]. FXR is not expressed in muscle but is expressed in white adipose at a very low level. It is noticed that *fxr*−/− mice had smaller adipocytes, and FXR agonist GW4064 treatment increased adipose differentiation and insulin-dependent glucose uptake in 3T3-L1 cells in vitro. Another study suggests that FXR agonist INT747 induced adipose differentiation via inducing the expression of adipocyte-related genes including C/EBP*α* and PPAR*γ* [51]. In addition to a role of FXR in adipose, two recent studies provided additional mechanism by which FXR may regulate peripheral glucose homeostasis. These studies revealed that FXR is also expressed in pancreatic *β* cells and positively regulates glucose-dependent insulin secretion [52]. It is suggested that FXR activation stimulates insulin gene transcription. On the other hand, FXR activation is associated with increased AKT phosphorylation and GLUT2 translocation to the cell membrane and thus enhances glucose uptake into pancreatic *β* cells and glucose-dependent insulin secretion. Intestine is another major FXR expressing tissue. A recent study showed that FGF15/19, expressed in the intestine and secreted into the blood circulation, acts as a postprandial factor that promotes glycogen synthesis, which may be an important mechanism controlling postprandial glucose metabolism [53]. It has been shown that serum FGF19 increases during postprandial period in humans, presumably due to increased bile acid signaling [34]. Therefore, identification of the regulatory role of FGF15/19 in postprandial glycogen synthesis provides a novel link between bile acid signaling and glucose metabolism. In addition to the nuclear receptor FXR-mediated effects, bile acids have been shown to directly activate hepatic AKT via a G*α*_i_ protein coupled receptor signaling pathway, which stimulated hepatic glycogen synthesis [54]. Recently, it was further demonstrated that bile acid activation of the G*α*_i_-AKT signaling cascade was involved in the bile acid induction of FXR and SHP and downregulation of gluconeogenic gene expressions in the liver [55]. In summary, these studies suggest that bile acid regulation of hepatic glucose metabolism involves complex crosstalk between FXR-dependent pathways and FXR-independent signaling pathways.

### 5.2. TGR5 and Glucose Metabolism

Bile acids also activate a cell surface G-protein coupled receptor TGR5, which is mainly expressed in the intestine, brown adipose, white adipose, and gallbladder. Low levels of TGR5 expression has also been detected in liver and skeletal muscle. Upon activation, TGR5 leads to intracellular cAMP production and PKA activation. Based on the ability to induce cellular cAMP production, taurolithocholic acid (TLCA) and LCA show highest potency in activating TGR5 with EC_50_ of 0.33 and 0.53 *μ*M, respectively, while DCA, CDCA, and CA (in rank order) activate TGR5 at higher 1–8 *μ*M concentrations [56]. It is suggested that in brown adipocytes, bile acid activation of TGR5-cAMP-PKA cascade results in induction of downstream deiodinase, fatty acid oxidation genes, and uncoupling proteins, which increase energy expenditure and promote weight loss [57]. As increased free fatty acid release and cytokine production associated with obesity clearly contribute to the development of insulin resistance, bile acids/TGR5 regulation of weight loss certainly could play a role in regulating glucose homeostasis. However, studies in *tgr*5−/− mice showed that neither under chow condition nor under high fat diet feeding condition did *tgr*5−/− develop obesity or hyperglycemia [58]. The high fat diet feeding effect on insulin sensitivity, as determined by insulin tolerance tests, also seemed to be gender-specific in *tgr*5−/− mice, with male showing impaired, but female showing improved insulin sensitivity [58]. The most potent endogenous ligand for TGR5 is TLCA. TLCA is highly toxic and once synthesized, is rapidly metabolized in the intestine and the liver. Under physiological conditions, liver efficiently extracts bile acids from the portal circulation, and bile acid concentration in the systemic circulation is very low. Because these primary and secondary bile acids activate TGR5 at a higher EC_50_, it is possible that TGR5 is not activated by physiological concentration of circulating bile acids outside of the entero-hepatic system. Thus, opposing to the clear pharmacological benefits of TGR5 activation, the physiological role of TGR5 in mediating bile acid signaling control of metabolic homeostasis needs to be further investigated. In addition to brown adipose, intestine is another major TGR5 expressing tissue. Using an enteroendocrine cell line STC-1, Katsuma et al. first demonstrated that bile acids stimulate glucagon like peptide-1 (GLP-1) production via TGR5 activation [59]. The pharmacological significance of this pathway was then demonstrated by a detailed study carried out by Thomas et al. [60]. These authors showed that administration of a potent TGR5 agonist INT777 raised intracellular ATP/ADP ratio and calcium influx, which leads to enhanced GLP-1 secretion from the intestine. GLP-1 is known to promote insulin secretion and thus regulate glucose homeostasis. Because GLP-1 mimetics and receptor agonists are currently under clinical development and have shown promise in improving glucose homeostasis in diabetes, bile acid-based TGR5 agonists may be a potential therapeutic to stimulate GLP-1 secretion in diabetic patients [61]. 

In contrast to these studies, bile acid sequestrants, which remove bile acids from the body by binding to bile acids in the intestine and prevent bile acids from being reabsorbed, have been shown to improve insulin sensitivity and lower fasting glucose in both men and several different experimental models [62]. Two studies conducted in rats have so far suggested that bile acid sequestrants may improve insulin sensitivity by increasing GLP-1 release [63, 64]. Although the molecular mechanism is still not clear, both studies suggested that such effect is likely bile acid receptor-independent. This is because both studies showed that administration of bile acid sequestrants significantly lowered serum bile acid levels, which was associated with decreased FXR activation in the liver and the intestine. Furthermore, it is shown that blocking intestine bile acid transport using SC-435, an apical sodium-dependent bile acid transport inhibitor, also lowered serum bile acid levels, but did not modulate insulin sensitivity or GLP-1 secretion. Thus, it is likely that bile acid sequestrants exert its effect by directly modulating cellular signaling in the intestine rather than by altering circulating bile acid levels or modulating bile acid pool.

## 6. Bile Acids and Lipid Metabolism

### 6.1. Bile Acids and Cholesterol Metabolism

It has been known for a long time that preventing bile acid reabsorption in the intestine by bile acid sequestration increases hepatic CYP7A1 and bile acid synthesis [65]. The resulting increase in hepatic cholesterol catabolism caused compensatory induction of LDL receptor (LDLR) and LDL cholesterol (LDL-C) uptake. Because of the activation of this liver pathway, cholestyramine has been used to effectively lowering serum cholesterol in human patients. Paradoxically, activation of FXR by its potent agonists, which repress hepatic bile acid synthesis, also decreased serum cholesterol in animal models [7]. In wild-type mice, activation of FXR is mainly associated with a reduction of HDL-C, while in hypercholesterolemic animal models, activation of FXR decreases both LDL-C and HDL-C. In vitro, FXR was shown to induce LDLR expression and repress PCSK9, an LDLR inhibitor [66]. However, activation of FXR still significantly decreased serum non-HDL cholesterol in *ldlr*−/− mice [67]. Furthermore, CDCA administration has been shown to raise serum LDL-C levels in humans. It remains to be determined whether activation of FXR will provide benefits in lowering LDL-C in men. 

FXR agonists have been shown to prevent atherosclerosis in various experimental models [68]. Serum HDL transports cholesterol from peripheral tissues to the liver for elimination and thus plays a critical role in reverse cholesterol transport and the development of atherosclerosis. However, the role of FXR in regulating HDL metabolism is still under debate because FXR inhibits the hepatic production of apolipoprotein A1 (ApoAI), a key structural component of HDL, and activation of FXR is associated with decreased serum HDL [69]. Nevertheless, a recent study showed that activation of FXR promotes reverse cholesterol transport in mice by inducing hepatic expression of scavenger receptor B1 (SR-B1) [70], which is suggested to play a role in both hepatic uptake of HDL-C and biliary secretion of free cholesterol [71]. A FXR binding site has been identified in the *SR-B1* gene promoter [72]. In a recent study, we demonstrated that stimulating de novo bile acid synthesis by transgenic expression of a *CYP7A1* gene in mouse liver prevented diet-induced hypercholesterolemia [6]. Different from CA feeding or FXR agonist administration, *Cyp7a1*-tg mice showed both increased hepatic cholesterol catabolism and bile acid signaling. Using this model, we demonstrated that bile acid activation of FXR induces hepatic expression of ABCG5 and ABCG8 through a common FXRE, which promoted biliary-free cholesterol secretion and fecal cholesterol loss. It is well known that cholesterol activation of LXR only induces mouse, but not human *CYP7A1* gene expression [73]. We also showed that cholesterol/LXR signaling only induced ABCG5 and ABCG8 in mice, but not in primary human hepatocytes [6]. These studies suggest that upon hepatic cholesterol accumulation, LXR may stimulate cholesterol catabolism or biliary cholesterol secretion in mouse livers, but not human livers. Thus, it is possible that bile acid/FXR/ABCG5/G8 pathway plays a more important role in maintaining hepatic cholesterol homeostasis in response to increased cholesterol levels in humans.

### 6.2. Bile Acids and Fatty Acid Metabolism

It has been known for a long time that serum bile acid and serum triglycerides are inversely correlated, suggesting that bile acid negatively regulates serum triglycerides [74, 75]. Current studies suggest that bile acids may lower serum triglycerides by repressing both hepatic triglyceride production/secretion and stimulating serum triglyceride clearance. In the liver, it is shown that bile acid activation of FXR repressed LXR-induction of SREBP-1 and its target genes in de novo lipogenesis by inducing the repressor SHP, which not only decreased hepatic fat accumulation, but also led to reduced hepatic VLDL secretion [76]. One study showing both LXR-dependent and FXR-dependent induction of hepatic lipogenesis by bile acid sequestrants administration supported this conclusion [68]. The finding that FXR represses microsomal triglyceride transfer protein (MTP) and thus hepatic VLDL secretion seems to provide additional support that the FXR/SHP pathway reduces hepatic triglyceride output [77]. Genetic knockout of *shp* in *ob/ob* mice increased MTP and VLDL secretion [78]. Diabetes and obesity are associated with increased hepatic VLDL output. Both increased fatty acid supply to the liver and hepatic insulin resistance may be involved. On the other hand, there are also studies showing that hepatic VLDL secretion was impaired in diabetic mouse models despite increased triglyceride output [79]. Furthermore, increased VLDL secretion in *shp* knockout mice seemed to be beneficial in reducing hepatic fat accumulation in *ob/ob* mice [78]. Thus, the bile acid regulation of hepatic VLDL secretion and its in vivo significance seem to be complex and may depend on the experimental conditions. Serum triglyceride is cleared after VLDL-triglyceride is hydrolyzed by lipoprotein lipase (LPL) and subsequently taken up by the peripheral tissues. It has been reported that obesity and diabetes are also associated with impaired peripheral triglyceride clearance, contributing to diabetic hypertriglyceridemia. Activation of FXR has been shown to induce apolipoprotein CII (ApoCII), which is an LPL activator, and repress apolipoprotein CIII (ApoCIII), which is an LPL inhibitor in the liver [80, 81]. Increasing ApoCII or decreasing ApoCIII stimulates LPL to hydrolyze triglycerides carried by VLDL, thus accelerates serum VLDL clearance upon FXR activation.

## 7. Conclusion

Extensive investigations conducted in the past decade have shown that bile acids are important regulators of glucose and lipid metabolism. The identification of bile acid-activated nuclear receptor FXR and cell surface G protein coupled receptor TGR5 has significantly advanced our understanding on how bile acid signaling regulates cellular metabolism in physiological and diseased conditions. The identification of these regulatory mechanisms also provided molecular basis for developing bile acid receptor agonists and receptor antagonists for treating human metabolic diseases. On the other hand, conflicting studies in the field are present, which not only reflects the complex nature of the bile acid signaling in regulation of whole body metabolism, but also implies the difference between physiological role and pharmacological role of bile acid signaling in metabolic control. Furthermore, studies that focus on the regulation of bile acid metabolism in diseased conditions, especially obesity and diabetes, are still insufficient. Future advances in the field are needed to improve our understanding in the bile acid control of metabolism, which is also critical in developing better drug therapies for the treatment of metabolic disorders.

## Figures and Tables

**Table 1:** FXR target genes and their function and lipid and glucose metabolism.

	Gene	Tissue	Regulation	Function
Bile acid metabolism	CYP7A1	liver	down	Encodes the rate-limiting enzyme in classic bile acid synthetic pathway
	BSEP	liver	up	Rate-limiting step in canalicular bile acid transport into the gallbladder
	NTCP	liver	down	Basolateral bile acid uptake into the hepatocytes
	OST*α*/*β*	intestine	up	Enterocyte basolateral bile acid secretion into the portal blood
	I-BABP	intestine	up	Intracellular bile acid transport
	FGF15/19	intestine	up	Bile acid synthesis inhibition
	SHP	liver	up	Bile acid synthesis inhibition

Glucose metabolism	PEPCK	liver	up	Gluconeogenesis
	FGF15/19	intestine	up	Stimulates glycogen synthesis, repress gluconeogenesis
	Insulin	pancreas	up	Glucose metabolism

Cholesterol metabolism	ApoA1	liver	down	HDL metabolism
	LDLR	liver	down	LDL uptake
	ABCG5/G8	liver	up	Biliary-free cholesterol secretion
	SRB1	liver	up	Hepatic HDL uptake, biliary cholesterol secretion
	PCSK9	liver	down	Induces LDL receptor degradation

Fatty acid metabolism	ApoC II	liver	up	LPL activator
	ApoC III	liver	down	LPL inhibitor
	SREBP1	liver	down	Lipogenesis

## Abbreviations

CA: Cholic acid
CDCA: Chenodeoxycholic acid
DCA: Deoxycholic acid
LCA: Lithocholic acid
CYP7A1: Cholesterol 7*α*-hydroxylase
CYP8B1: Microsomal sterol 12*α*-hydroxylase
CYP27A1: Mitochondrial 27-hydroxylase
CYP7B1: Oxysterol 7*α*-hydroxylase
MCA: Muricholic acids
ABC: ATP-binding cassette
BSEP: Bile salt export pump
SPGP: Sister of P-glycoprotein
PFIC: Progressive familial intrahepatic cholestasis
MDR: Multidrug resistance
NTCP: Na^+^-dependent taurocholate transporter
OATP: Organic anion transporter
ASBT: Apical sodium-dependent bile salt transporter
I-BABP: Intestinal bile acid binding protein
OST: Organic solute transporter
FXR: Farnesoid X receptor
SHP: Small heterodimer partner
LRH-1: Liver-related homologue-1
FGF15: Fibroblast growth factor 15
PEPCK: Phosphoenolpyruvate carboxykinase
GLP-1: Glucagon-like peptide-1
LDL: Low density lipoprotein
ApoA1: Apolipoprotein A1
SR-B1: Scavenger receptor B1
MTP: Microsomal triglyceride transfer protein
LPL: Lipoprotein lipase
ApoCII: Apolipoprotein CII
ApoCIII: Apolipoprotein CIII
VLDL: Very low density lipoprotein.
